# Clash of the Invaders: Competition Dynamics of *Bromus tectorum* and *Ventenata dubia* in an Addition Series Study

**DOI:** 10.1002/ece3.71458

**Published:** 2025-05-19

**Authors:** Lilly Sencenbaugh, Bruce D. Maxwell, Lisa J. Rew

**Affiliations:** ^1^ Land Resources and Environmental Sciences Montana State University Bozeman Montana USA

## Abstract

Competitive interactions between co‐occurring invasive species can have detrimental impacts on native communities and cause counter‐effective responses to management. Targeted removal of one invader may allow for the release of a subdominant invader, causing a secondary invasion. The goal of this research was to elucidate competitive dynamics between *Bromus tectorum* and *Ventenata dubia,* two invasive winter annual grasses found in the western United States*.* We quantified the impacts of (1) intraspecific competition on *B. tectorum* and *V. dubia* as the density of conspecifics increased and (2) interspecific competition between the two at varying proportions. The two species were grown at increasing densities and proportions (addition series) over 10 weeks in a greenhouse. Aboveground biomass was harvested and weighed. We derived the intraspecific and interspecific competitive effects on each species with a nonlinear analysis and used these coefficients to determine relative competitive ability (RCA). Both species were impacted by interspecific competition and intraspecific competition. More conspecifics were required to cause a decline in both species' biomass relative to the number of allospecifics that caused the same response. Interestingly, the number of allospecific individuals to imposed an impact was similar. The RCA values for both species were < 1, which indicated that interspecific competition had a greater influence on both species than intraspecific competition. This suggests that the replacement of *B. tectorum* by *V. dubia* is unlikely to be caused by aboveground competition alone. However, there are differences in germination timing between the two species; both germinate in the fall, but *V. dubia* also germinates in the spring. Management that targets fall‐germinating individuals may reduce *B. tectorum* and fall‐germinating *V. dubia* but not impact spring‐germinating *V. dubia,* which may release these individuals from competition. Understanding the competitive interactions between these species provides insight into invasive species impacts and management.

## Graphical abstract

Competitive interactions between co‐occurring invasive species can have detrimental impacts on native community composition and cause countereffective responses to management. The goal of this research was to elucidate competitive dynamics between *Bromus tectorum* and *Ventenata dubia*, two invasive winter annual grasses found in the western United States by quantifying the impacts of intraspecific and interspecific competition between the two at varying proportions. We found that both species were most impacted by interspecific competition, though neither had an advantage over the other.*B. tectorum* has a sl

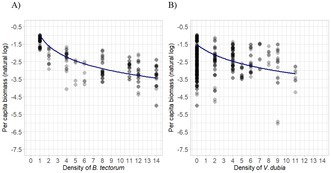

## 1 Introduction

Invasive species are overwhelmingly found in habitats where other invaders are also present (Kuebbing et al. 2013; Pearson et al. 2016b; Brandt et al. 2023), where they threaten native plant diversity (Vilà et al. 2011) and can have negative impacts on ecosystem function and composition (Kuebbing and Nuñez 2015). However, competitive interactions between different invader species, or “invasional interference” (Yang et al. 2011), can limit their population density and spread due to competitive limitations for resources (Kuebbing and Nuñez 2015; Rauschert and Shea 2012). Under invasive interference circumstances, impacts on the entire community may be less intense because the invader populations could limit themselves (Brandt et al. 2023; Rauschert and Shea 2012; Rauschert and Shea 2017). Understanding the competitive interactions between co‐occurring species is crucial to providing insight into community assembly dynamics. This is especially so with co‐occurring invaders that are particularly invasive, as their interactions can enhance detrimental impacts on native community composition and cause countereffective responses to management (Kuebbing et al. 2013; Kuebbing and Nuñez 2015).

A major dilemma in the restoration of native communities is that the targeted removal of a dominant invasive species can allow for competitive release of co‐occurring nontarget invasive species, a phenomenon known as secondary invasion (Kuebbing and Nuñez 2015; Pearson et al. 2016b). Dominant invaders place competitive pressures on subdominant invasive species, but their removal decreases invasional interference and can cause the nontarget invaders to increase in density or space (Pearson et al. 2016b; Shen et al. 2023; Torres et al. 2023). In a meta‐analysis of 38 secondary invasion studies, Pearson et al. (2016b) found reductions in target invader density were related to increases in the abundance of nontarget invaders and corresponded with minor increases in native plants. Particularly, they found that more targeted weed control methods (hand pulling, cutting, biocontrol, and targeted herbicides) elicited a stronger response in secondary invasion (Pearson et al. 2016b). There is a body of literature that documents secondary invasions following management efforts in semi‐natural and natural settings (Brigham et al. 2024; Hata et al. 2024; Larson and Larson 2010; Lesica and Hanna 2009; Rauschert and Shea 2012; Reid et al. 2009; Shen et al. 2023; Skurski et al. 2013; Torres et al. 2023). These studies generally found that most often the secondary invader is different from the original invader in phenology or growth form (Butler and Wacker 2010; Larson and Larson 2010; Lesica and Hanna 2009; Ortega and Pearson 2011; Pearson et al. 2016b; Skurski et al. 2013). This may be due to similar invaders being more likely to be affected by the same management strategy, whereas invaders with different traits will escape the impacts of management and take advantage of the removal of the other invader (Pearson et al. 2016b). An example is the secondary invasion of the non‐native annual grass *Bromus tectorum* L. (downy brome, cheatgrass) following removal of the non‐native perennial forb *Centaurea stoebe* L. (spotted knapweed) (Ortega and Pearson 2011; Skurski et al. 2013). However, co‐occurring invaders with similar phenology or growth forms are not uncommon (DiTomaso et al. 2010; Northam and Callihan 1994; Prather and Burke 2011; Rauschert and Shea 2012), for example, *B. tectorum* is found co‐occurring with a myriad of other non‐native annual grasses like *B. squarrosus* L. (corn brome), *Ventenata dubia* (Leers) Coss. (ventenata, North Africa grass), and *Taeniatherum caput‐medusae* (L.) Nevski (medusahead) (DiTomaso et al. 2010; Northam and Callihan 1994; Wallace et al. 2015). But secondary invasion of a non‐native annual grass following the removal of another non‐native annual grass has not been conclusively demonstrated. From possible observations of interference in the field, finer‐tuned competition experimentation may elucidate patterns and processes with greater clarity.

Our interpretation of competition is influenced by the ways we structure our competition studies (Cousens 1991; Leon et al. 2023). Pairwise studies of plant competition are helpful to clarify fine‐scale impacts of species on each other. However, frequently used experimental designs account for only the density of plants (additive) or proportion (replacement series), which limits our understanding of intraspecific and interspecific density dependence and competition. A design that accounts for both intraspecific and interspecific density dependence is the addition series (Connolly et al. 2001), which includes a range of replacement series designs at increasing densities, altering density and proportion simultaneously (Cousens 1991; Firbank and Watkinson 1985; Inouye 2001; Spitters 1983). Measurements are taken of productivity (e.g., biomass, cover) on an individual plant basis, allowing quantification of per capita impacts of density and proportion (Firbank and Watkinson 1985; Radosevich et al. 2007; Spitters 1983). Using relative competitive abilities analyses from addition series data offers a more in‐depth understanding of competitive interference than other approaches. The addition series may provide a more realistic picture of how species compete with one another in a biculture, providing a first‐principle assessment for estimating interaction outcomes. Execution of the design and analysis is often complicated and time‐consuming (Levine et al. 2017; Radosevich et al. 2007). However, using the addition series design on co‐occurring invasive species could provide insight into effective management scenarios and the future of invasions.

The proliferation of invasive non‐native annual grasses is a critical problem in rangelands of the American West (DiTomaso 2000). The invasive annual grass, *B. tectorum* , has been present for 150 years and has a noticeable inverse relationship with the density and cover of native perennial grasses (De Stefano et al. 2024; Mack 1981; Reisner et al. 2013). *Ventenata dubia* is a newer invader to the region, first detected in the 1950s (Barkworth et al. 1993; Fryer 2017), and has also been observed to negatively impact native species (Jones et al. 2018; Jones et al. 2020; Wallace et al. 2015). Both species are cool‐season C3 winter annual grasses that germinate in the fall (Fryer 2017; Mack 1981; Wallace and Prather 2016), although observations have been made of *V. dubia* germinating in the spring, particularly in rangelands (Wallace et al. 2015). These species are both found in the western United States, and concerns have been raised that *V. dubia* is spreading into areas that were once dominated by *B. tectorum* (Barkworth et al. 1993; Northam and Callihan 1994; Prather and Burke 2011). *Bromus tectorum* is primarily an issue in sagebrush steppe and other drier regions such as the Great Basin Desert, but predictions using future climate change scenarios indicate improved habitat suitability in northerly and easterly directions (Bradley 2009; Taylor et al. 2014). Further, *B. tectorum* has been identified as the invasive species with the highest impact in the Intermountain West (Pearson et al. 2016a) While *V. dubia* is currently an issue in the inland Pacific Northwest (Jones et al. 2020; Wallace et al. 2015), its suitable habitat is also expected to change with heating and drying summer conditions (Adhikari et al. 2022; Chang et al. 2023; Nietupski et al. 2024), overlapping areas that are expected to also become more suitable for *B. tectorum* .

As these two species are problematic invaders individually, it is vital that we understand the outcomes of their interaction to make effective management decisions. The goal of this study was to determine the intraspecific and interspecific competitive dynamics of *B. tectorum* and *V. dubia* . We sought to quantify differences in intraspecific and interspecific competition of these species along an increasing density gradient to understand if aboveground competition is the driving mechanism by which *V. dubia* could replace *B. tectorum* in the field. To do this, we evaluated individual plant aboveground biomass when grown in an addition series and determined the coefficients of intraspecific and interspecific competition, and at what densities aboveground per capita biomass began to be reduced. We used these coefficients to quantify the relative competitive ability. We hypothesize that both species will be impacted similarly by intraspecific density, a common finding in annual grass species, but that *B. tectorum* will have a greater interspecific impact on *V. dubia*, as *B. tectorum* has demonstrated greater growth than *V. dubia* when compared in greenhouse settings (Bansal et al. 2014; James 2008). This could mean that under field conditions, *V. dubia* could be released from competition by *B. tectorum,* leading to a secondary invasion. Our results could be used to inform future management decisions under different density scenarios where these species co‐occur.

## 2 Methods

### 2.1 Experimental Design

**FIGURE 1:**
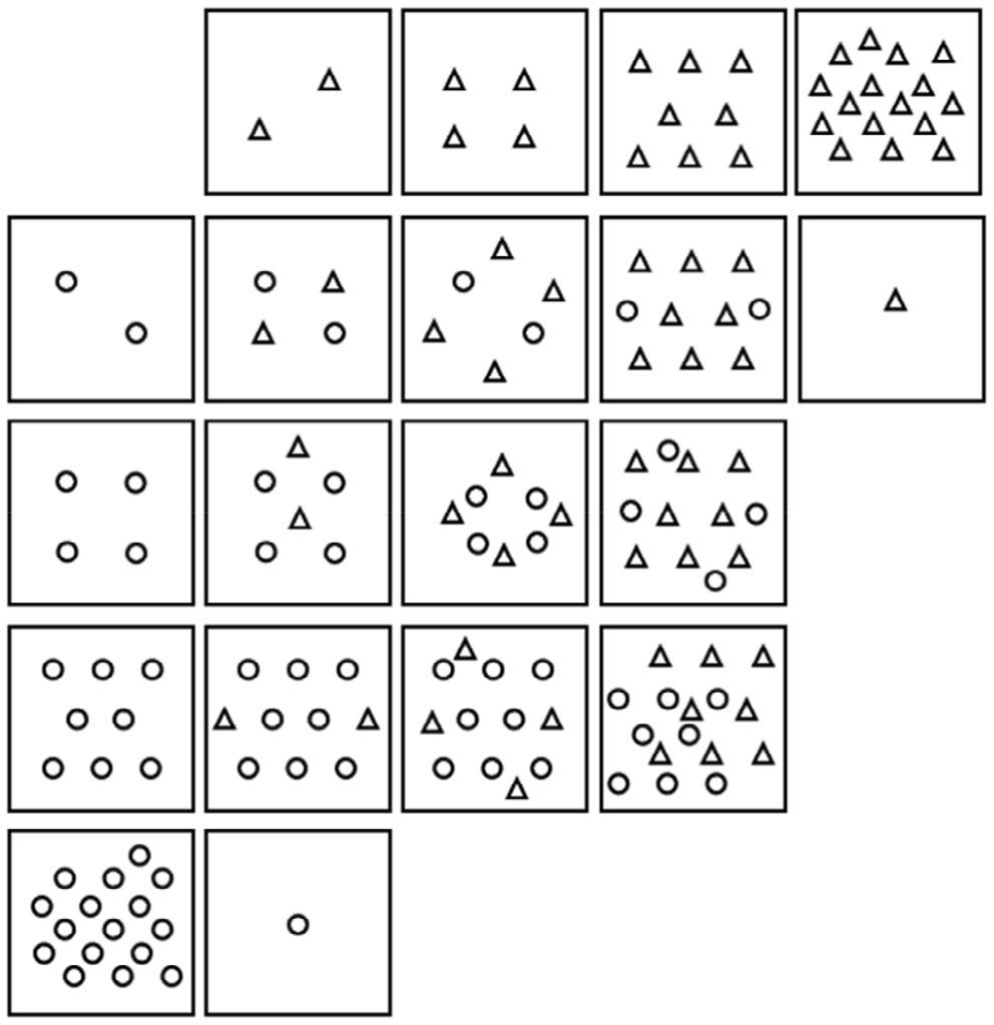
Plant layout in pots for each competition arrangement, where ο represents *Bromus tectorum* and ∆ represents *Ventenata dubia*. This design was an addition series as proposed by Spitters (1983) and Firbank and Watkinson (1985).

*Bromus tectorum* and *V. dubia* were planted into four repetitions of an addition series matrix (Figure 1). Five density levels were planted for each species (1, 2, 4, 8, and 16), where the middle densities (2, 4, and 8) were grown in monoculture and mixture, and the lowest and highest densities (1 and 16) were in monoculture only, although no pots maintained the desired 16 plants to the end of the experiment. Densities were chosen after reviewing other competition studies that included one or more of these species so that middle densities were high enough to elicit a response (Harvey et al. 2020; Larson et al. 2018).

*Bromus tectorum* seeds were collected near Norris, MT, from Red Bluff Research Ranch (N 45°33′1.85121″, W 111°39′30.62804″). *Ventenata dubia* seeds were sourced from near Bozeman, MT (N 45°45′32.3″, W 111°08′39.3″); Lodge Grass, MT (N 45°16′17.4″, W 107°35′16.9″); and Missoula, MT (N 46°53′55.6″, W 113°56′58.3″). Pots (15 cm diameter and 11 cm deep, 1767 cm^3^) were filled with pasteurized (70°C for 60 min) potting soil that was equal parts loam soil, washed concrete sand, and Canadian Sphagnum peat moss. Seeds were planted into the pots to achieve the desired densities according to the experimental design. The study was run for 10 weeks in a Montana State University Plant Growth Center (N 45°40′05.3″, W 111°03′12.0″) greenhouse (light/dark/18°C–29°C/10°C–23°C, MVR1000/C/U multivapor bulbs GE Lighting) and watered every other day. At the end of 10 weeks, individual plants were harvested for aboveground biomass. Samples were placed in coin envelopes and dried for 48 h at 37°C. Plants were weighed for biomass to accuracy of 0.001 g.

### 2.2 Statistical Methods

All statistical analyses were performed in R (R Core Team 2024). Analysis of each species' biomass was performed using methods after Spitters (1983) and Firbank and Watkinson (1985). We used the monoculture density data to analyze the intraspecific competition, and density and proportion data for interspecific competition: for both, we used nonlinear regression. These analyses provide coefficients for intraspecific and interspecific competition, which were then used to determine the density thresholds at which intraspecific and interspecific competition caused significant impact on per capita biomass. We used the coefficients of intraspecific and interspecific competition to determine the relative competitive ability (Evans et al. 1991; Roush et al. 1989).

We first determined mean biomass per plant when grown along a density gradient in monoculture. Where mean yield per plant (w) can be described by the equation:

(1) $$w=w_{m}{\left(1+\text{aN}\right)}^{-b}$$

where $w_{m}$ was the mean biomass of individuals grown alone, a was the area required to reach the biomass $w_{m}$, N was the total density of plants at harvest, and b was thought to be the mean species‐specific resource use efficiency (Firbank and Watkinson 1985). We then determined the mean biomass per plant when grown at increasing density and different proportions. Where mean biomass per plant of *B. tectorum* ($w_{B})$ or *V. dubia* ($w_{V}$) can be described by the equations:

(2) $$w_{B}=w_{\mathrm{mB}}{\left(1+\beta_{B}\;\left(N_{B}+\alpha_{\mathrm{BV}}N_{V}\right)\right)}^{-b_{B}}$$

(3) $$w_{V}=w_{\mathrm{mV}}{\left(1+\beta_{V}\;\left(N_{V}+\alpha_{\mathrm{VB}}N_{B}\right)\right)}^{-b_{V}}$$

where $w_{\mathrm{mB}}$ was the mean biomass of *B. tectorum* individuals grown alone, $\beta_{B}$ was the slope of *B. tectorum* intraspecific competition, $\beta_{V}$ was the slope of *V. dubia* intraspecific competition, $\alpha_{\mathrm{BV}}$ was the competition coefficient of the impact of *V. dubia* on *B. tectorum*, $\alpha_{\mathrm{VB}}$ was the competition coefficient of the impact of *B. tectorum* on *V. dubia*, $N_{B}$ was the density of *B. tectorum* at harvest, $N_{V}$ was the density of *V. dubia* at harvest, and $b_{B}$ was thought to be the mean species‐specific resource use efficiency of *B. tectorum* and $b_{V}$ for *V. dubia* .

We first derived α and b from Equation (1) using nonlinear least squares methods for our two species when grown in monocultures. We performed diagnostic tests and, due to heteroscedasticity of our data, filtered extreme outliers (outliers, likely due to mistakes during data recording or collection, defined as values outside of 1.5 times the interquartile range, i.e., outside of 95% of observations; Table 1). Models were then refit with a natural log transformation. The final natural log‐transformed model for monoculture analysis was as follows:

(4) $$\ln\left(w\right)=\ln\left(w_{m}\right)-b*\ln\left(1+\mathrm{\alpha N}\right)$$

**TABLE 1:** Number of observations and outliers removed from each experiment.

Species	Experiment	Total observations	Outliers removed	Final observations
*Bromus tectorum*	Monoculture	225	30	195
	Biculture	420	30	390
*Ventenata dubia*	Monoculture	236	26	210
	Biculture	464	56	408

The final natural log‐transformed models for the biculture analyses were as follows:

(5) $$\ln\left(w_{B}\right)=\ln\left(w_{\mathrm{mB}}\right)-b_{B}*\ln\left(1+\beta_{B}\left(N_{B}+\alpha_{\mathrm{BV}}N_{V}\right)\right)$$

(6) $$\ln\left(w_{V}\right)=\ln\left(w_{\mathrm{mV}}\right)-b_{V}*\ln\left(1+\beta_{V}\;\left(N_{V}+\alpha_{\mathrm{VB}}N_{B}\right)\right)$$

The parameters α and b from Equation (4) were used in Equations (5) and (6), where we were able to determine the intraspecific and interspecific competition coefficients for each species (β and α, respectively).

To determine the density thresholds at which species *B. tectorum* and species *V. dubia* demonstrated 50% and 75% reductions in biomass, we derived these thresholds from Equations (5) and (6). Thresholds were calculated using the estimated parameters of the model and back transformed. Confidence intervals for the thresholds were obtained by propagating uncertainty in the parameter estimates through the back transformation process. Following the determination of the intraspecific and interspecific competition coefficients for each species, we determined the relative competitive ability (Roush et al. 1989; Spitters 1983) using the equations:

(7) $$R{\mathrm{CA}}_{B}=\frac{\beta_{B}}{\alpha_{\mathrm{BV}}}$$

(8) $$R{\mathrm{CA}}_{V}=\frac{\beta_{V}}{\alpha_{\mathrm{VB}}}$$

where ${\mathrm{RCA}}_{B}$ is the relative competitive ability of *B. tectorum*, and ${\mathrm{RCA}}_{V}$ is the relative competitive ability of *V. dubia* . Relative competitive ability can be interpreted as the density equivalence between species. For example, if *RCA*_*B*_ is 0.5, it would take two individuals of *V. dubia* to equal one individual's biomass of *B. tectorum* .

## 3 Results

When grown in monoculture, *B. tectorum* biomass ranged from 0.001 to 0.377 g with a mean per capita biomass of 0.113 g (*n* = 195). After 10 weeks of growth, mean biomass when grown in monoculture at increasing density can be best described by the model:

(9) $$\ln\left(w\right)=\ln\left(0.113\right)-0.96848*\ln\left(1+9.18040N\right)$$

Pseudo‐*r* ^*2*^ = 0.56; *n* = 195.

**FIGURE 2:**
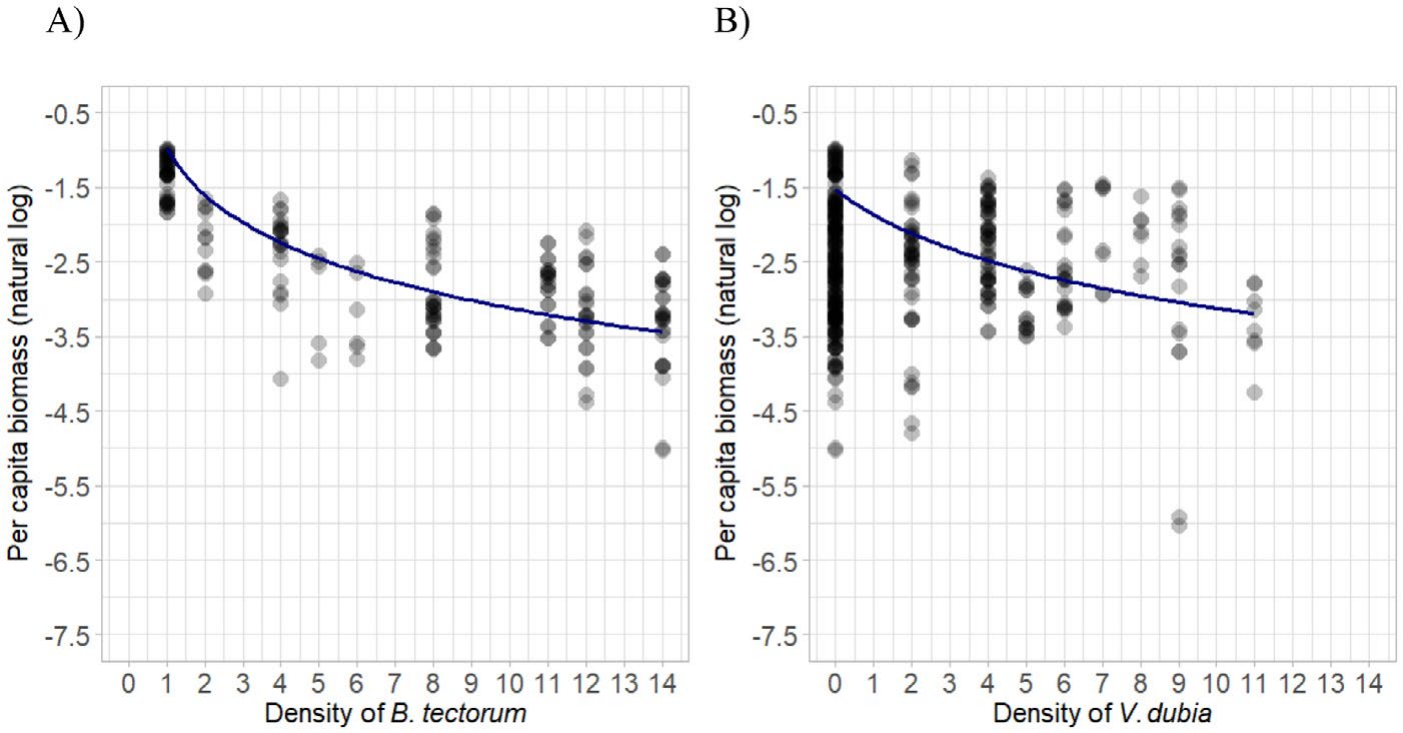
Natural log transformed per capita biomass of *Bromus tectorum* after 10 weeks relative to the density of either (A) *B. tectorum* individuals or (B) *V. dubia* individuals in a controlled environment. Lines represent predicted model fit for mean biomass when grown in (A) intraspecific competition (Equation (9)), and (B) interspecific competition with *V. dubia* (Equation (10)).

The area required to reach maximum biomass is estimated to be 9.180 cm^2^ (~3 cm diameter), and the resource use efficiency was 0.968, which was used for the calculation of intraspecific and interspecific competition (Equation (5); Figure 2A). While in competition with *V. dubia* , the mean per capita biomass was 0.108 g (*n* = 390). Mean biomass when grown in competition with *V. dubia* at increasing densities and proportions can be best described by the model:

(10) $$\ln\left(w_{B}\right)=\ln\left(0.10768\right)-0.96848*\ln\left(1+0.13090\left(N_{B}+5.6387N_{V}\right)\right)$$

Pseudo‐*r* ^*2*^ = 0.43; *n* = 390.

There was evidence of a negative impact of *B. tectorum* density on per capita *B. tectorum* biomass ($\beta_{B}=0.131,\;p=0.013$; Table 2; Figure 2A), and of an impact of *V. dubia* on *B. tectorum* biomass ($\alpha_{\mathrm{BV}}=5.639,\;p=0.021;$ Table 2; Figure 2B).

**TABLE 2:** Model output for Equation (10), which modeled the natural log biomass of *Bromus tectorum* given intraspecific ($\beta_{B}$) and interspecific ($\alpha_{\mathrm{BV}}$) competition.

Parameter	Estimate	Standard error	*t* value	*p*
$\beta_{B}$	0.1309	0.0524	2.4960	0.0130
$\alpha_{\mathrm{BV}}$	5.6387	2.4357	2.3150	0.0211

**TABLE 3:** Model output for Equation (12), which modeled the natural log biomass of *Ventenata dubia* given intraspecific ($\beta_{V}$) and interspecific ($\alpha_{\mathrm{VB}}$) competition.

Parameter	Estimate	Standard error	*t* value	*p*
$\beta_{V}$	0.5751	0.0472	12.1770	< 0.0001
$\alpha_{\mathrm{VB}}$	1.8818	0.3410	5.5190	< 0.0001

Relative competitive ability of *B. tectorum* was 0.023, in which 1 *B. tectorum* plant and < 1 *V. dubia* have equal influence on per capita biomass of *B. tectorum*. Intraspecific competition led to a 50% decline in *B. tectorum* biomass at a density of eight plants, where a 75% decline was estimated to occur at a density of 23 plants, although there was high variability. Interspecific competition led to a 50% decline in *B. tectorum* biomass at a density of one *V. dubia* plant, where a decline of 75% was detected at a density of four *V. dubia* plants (Table 4).

**TABLE 4:** Estimated number of plants (either *Bromus tectorum* or *Ventenata dubia* ) where a decrease in per capita biomass was detected to be 50% or 75%.

Species	Experiment	50% decline in biomass	95% confidence interval	75% decline in biomass	95% confidence interval
*Bromus tectorum*	Intraspecific	7.64272	4.22826, 29.48033	22.92816	12.68479, 88.44098
	Interspecific	1.35538	0.18389, 9.74081	4.06615	0.55167, 29.22244
*Ventenata dubia*	Intraspecific	1.74337	1.48237, 2.06506	5.23010	4.44711, 6.19518
	Interspecific	0.92698	0.55717, 1.62201	2.78097	1.67150, 4.86604

When grown in monoculture, *V. dubia* biomass ranged from < 0.001 to 0.114 g, where the mean per capita biomass was 0.028 g (*n* = 210). Mean biomass when in monoculture at increasing density can best be described by the model:

(11) $$\ln\left(w\right)=\ln\left(0.02808\right)-0.96730*\ln\left(\;1+222.14000N\right)$$

Pseudo‐*r* ^*2*^ = 0.18; *n* = 210.

**FIGURE 3:**
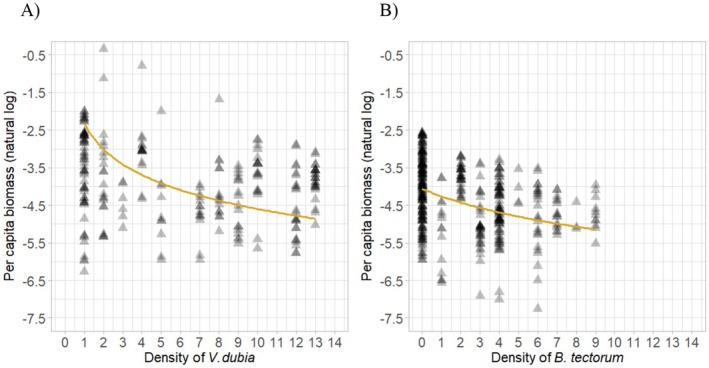
Natural log transformed per capita biomass of *Ventenata dubia* after 10 weeks relative to the density of either (A) *V. dubia* individuals or (B) *B. tectorum* individuals in a controlled environment. Lines represent predicted model fit for mean biomass when grown in (A) intraspecific competition (Equation (11)), and (B) interspecific competition with *B. tectorum* (Equation (12)).

The area required to reach maximum biomass was 222.140 cm^2^ (~17 cm diameter), where the resource use efficiency was 0.967, which was used for the calculation of intraspecific and interspecific competition (Equation (6); Figure 3A). When grown in a mixture with *B. tectorum*, the mean per capita biomass was 0.018 g (*n* = 408). Biomass of *V. dubia* when grown in competition with *B. tectorum* at varying densities and proportions can be best described by the model:

(12) $$\ln\left(w_{V}\right)=\ln\left(0.01803\right)-0.96730*\ln\left(1+0.57514\;\left(N_{V}+1.88184\;N_{B}\;\right)\right)$$

Pseudo‐*r* ^*2*^ = 0.36; *n* = 408.

There was strong evidence of a negative impact of increasing *V. dubia* density on *V. dubia* biomass ($\beta_{V}=0.575,\;p\;<0.001;$ Table 3; Figure 3A). There was also strong evidence of a negative impact of *B. tectorum* competition on *V. dubia* biomass ($\alpha_{\mathrm{VB}}=1.882,$ p<0.001; Table 3; Figure 3B), where increasing density of *B. tectorum* caused a decrease in *V. dubia* biomass.

Relative competitive ability of *V. dubia* was 0.306, in which 1 *V. dubia* plant and < 1 *B. tectorum* plant have equal influence on the per capita biomass of *V. dubia*. Intraspecific competition led to a 50% decline in biomass at a density of two *V. dubia* plants, where a 75% decline was detected at a density of five plants. Interspecific competition led to a 50% decline in *V. dubia* biomass at a density of one *B. tectorum* plant, where a 75% decline in biomass was detected at a density of three *B. tectorum* plants (Table 4). The difference in interspecific competition was not statistically different between the two species.

## 4 Discussion

Both species were more strongly impacted by interspecific competition than intraspecific competition, though neither species had an advantage over the other. We found that both reduced the other's biomass with similar number of individuals, ~1 for a 50% reduction in biomass and ~3.5 individuals for a 75% reduction, as the confidence intervals were overlapping. *Bromus tectorum* individuals were heavier than *V. dubia* , and mean weight did not differ much with intraspecific or interspecific competition. In contrast, *V. dubia* was half the weight when in interspecific versus intraspecific competition. Intraspecific competition impacted each species, except that *V. dubia* had a stronger impact on itself (conspecifics), with only two individuals required to reduce its own biomass by 50% compared with eight individuals for *B. tectorum* . The finding that interspecific competition was stronger than intraspecific is the opposite of the pattern which has been termed the modern coexistence theory (Barabás et al. 2018; Chesson 2000), which posits coexistence occurs when intraspecific competition is a greater driving factor than interspecific competition (Barabás et al. 2018; Chesson 2000; Chesson 2003). However, the finding that the two species impacted each other similarly could also allow them to co‐occur.

Predicted climate change scenarios in the American West correspond to increased habitat suitability for *V. dubia*, particularly in grasslands and agricultural lands (Adhikari et al. 2022; Nietupski et al. 2024) and a shift of *B. tectorum* into Montana and Wyoming (Bradley 2009; Taylor et al. 2014). If realized, these shifts will mean greater overlap between the two species. Current and potentially greater future interaction between *B. tectorum* and *V. dubia* needs to be considered in the management context. However, Harvey et al. (2020) grew *B. tectorum* and *V. dubia* in competition in a replacement series design under current‐day and projected 2100 CO_2_ and temperature. They found no competitive impacts under current climate and CO_2_, but *B. tectorum* had smaller biomass under the projected CO_2_ and temperature; the latter pattern was also observed in another study (Larson et al. 2018). *Ventenata dubia* had greater root‐to‐shoot ratios than *B. tectorum* , especially in the projected CO_2_ and temperature treatment, which may indicate *V. dubia* has greater water use efficiency, a trait that may give a competitive advantage in the field and under changing climate (Harvey et al. 2020). It is possible that this study did not grow *B. tectorum* and *V. dubia* at a high enough density for competition to be discernible via per capita biomass, as their density was a total of four plants per pot (10 × 10 × 12.5 cm, 1250 cm^3^) (Harvey et al. 2020), where we found only a 50% decline in per capita biomass of *V. dubia* at a density of 1 *B. tectorum* plant, compared with a 75% decline at five plants (although it should be noted their pot volume was roughly three‐quarters of ours). In determining the densities where competitive impacts are noticeable, we can make predictions about when invasional interference between these species may occur in the field. While we found little difference in aboveground interspecific competition between these species, studies suggest a changing climate may alter this interaction, as well as belowground interactions, in favor of *V. dubia* , especially as more habitats become suitable for *V. dubia* (Adhikari et al. 2022; Harvey et al. 2020; Wallace et al. 2015) and the regions they co‐occur in increase.

Experimentation to elucidate competitive relationships between species can provide useful information toward invasive species management. Manipulating the competitive dynamics between crops and weeds is a common approach in agroecosystems (Harker and O'Donovan 2017; Menalled et al. 2016), for example, by choosing crop cultivars with competitive traits (Andrew et al. 2015; O'Donovan et al. 2000) or through increased seeding rates (Harker et al. 2003; O'Donovan et al. 2000), but has been less regularly applied to rangelands or natural systems. In grassland systems invaded by non‐native annual grasses, there has been mixed success in combining herbicide treatments with restoration seeding to increase native perennial grasses (Link et al. 2017; Mangold et al. 2015; Monaco et al. 2017). This integrated approach has been suggested to prevent secondary invasion, as the newly seeded native species should fill the gaps left by the removed invader (DiTomaso 2000; Krueger‐Mangold et al. 2006). Relationships between annual and perennial grass species are influenced by developmental stage, where whichever species is more established, usually the perennial species from previous years' growth, will have a greater influence (Larson et al. 2018; Orloff et al. 2013). *Ventenata dubia* , however, has demonstrated invasion of previously uninvaded and low productivity systems regardless of resident vegetation type (Tortorelli et al. 2020; Tortorelli et al. 2022a). Further, Tortorelli et al. (2022b) found that experimental removal of neighboring plant biomass did not impact the spread of *V. dubia* along a productivity gradient; rather, *V. dubia* invasion seems more responsive to abiotic site conditions. Contrastingly, intact plant communities are often resistant to invasion by *B. tectorum*, where invasion tends to occur following disturbance that creates large quantities of bare ground (Chambers et al. 2016; Seipel et al. 2018). Both these non‐native annual grasses germinate in the fall, where perennial grasses most often germinate in the spring in this region and are senesced in the fall; this timing gives the non‐natives an advantage at the seedling stage. Further, the germination timing of the two non‐natives is slightly offset, with earlier germinating individuals of *B. tectorum* at a competitive advantage over later germinating individuals of *V. dubia* due to earlier resource acquisition (i.e., priority effect) (Ploughe et al. 2020). However, there is also evidence of *V. dubia* germinating in the spring (Wallace et al. 2015). These small differences in germination timing could be instrumental in allowing secondary invasion of *V. dubia* .

Managing co‐occurring invasive species in a way that avoids a secondary invasion is a challenge. A dichotomous guide to management actions for avoiding or mitigating the risk of secondary invasion is proposed by Pearson et al. (2016b), where it is suggested that if the potential secondary invaders are sensitive to the intended management tool, then that tool should be used, as it will address both the primary and secondary invader. In semi‐arid rangelands, the use of pre‐emergent herbicides applied in the fall can be effective at managing these invasive annual grass species to a similar degree while minimizing impact on the desired native perennials because they are senesced and thus uptake minimal herbicide (Clark et al. 2019; Elseroad and Rudd 2011; Koby et al. 2019; Mangold et al. 2013; Morris et al. 2009; Wallace and Prather 2016). In places where these species are co‐occurring, herbicide management in the fall would control both species, but early application could lead to a higher proportion of *B. tectorum* being removed due to differences in germination timing. This could cause a release of *V. dubia* , which, when combined with spring germinating cohorts that would not compete with *B. tectorum*, would lead to a secondary invasion of *V. dubia* . It should be noted that herbicide that has soil residual activity that lasts until the spring germination period may be effective at targeting both fall and spring germination of *V. dubia* (Koby et al. 2019; Sebastian et al. 2017).

Our experimental results, although conducted under highly controlled conditions, suggest that where feasible, management in areas that contain both *B. tectorum* and *V. dubia* should use techniques that impact both species equally *and* capture spring‐emerging *V. dubia* . While in many places there is an observed transition from *B. tectorum* to *V. dubia*, it is unlikely that it is caused exclusively by aboveground competitive effects. Instead, it may be that the removal of *B. tectorum* with management is creating open space and available resources that *V. dubia* is able to take advantage of before native perennial species emerge or start their spring growth. Insight into the competitive interactions between these co‐occurring invasive species provides vital information to inform management and how the invasion may change in the future. As our climate continues to change, new species will be interacting and new management responses will be needed: conducting an additive design study would help inform us of the patterns of these new interactions.

## Author Contributions

**Lilly Sencenbaugh:** conceptualization (lead), data curation (lead), formal analysis (lead), methodology (equal), writing – original draft (lead), writing – review and editing (equal). **Bruce D. Maxwell:** conceptualization (equal), formal analysis (supporting), methodology (equal), writing – review and editing (equal). **Lisa J. Rew:** conceptualization (equal), formal analysis (equal), methodology (equal), supervision (equal), writing – review and editing (equal).

## Conflicts of Interest

The authors declare no conflicts of interest.

## Data Availability

The data that support the findings of this study are available from the online repository: https://github.com/lil‐hammer6/competition‐bromus‐tectorum‐ventenata‐dubia.
